# Atypical Polypoid Bladder Lesion Mimicking Urothelial Carcinoma in a Young Male: A Case Report About a Possible Autoimmune-Triggered Granulomatous Inflammation With Secondary Colonization

**DOI:** 10.7759/cureus.98879

**Published:** 2025-12-10

**Authors:** Severin P Hofmann, Uwe Bieri, Franz von Stauffenberg, Stefan Schneider, Gad Singer, Michael Greiner, Lukas John Hefermehl

**Affiliations:** 1 Department of Urology, Kantonsspital Baden, Baden, CHE; 2 Department of Urology, Universitätsspital Zürich, Zurich, CHE; 3 Department of Rheumathology, Rheumazentrum Aarau, Aarau, CHE; 4 Department of Pathology, Kantonsspital Baden, Baden, CHE; 5 Department of Infectious Disease, Kantonsspital Baden, Baden, CHE

**Keywords:** gross hematuria, mimicking urothelial carcinoma, polypoid bladder lesion, secondary microbial colonization, vasculitis of the bladder

## Abstract

Gross hematuria is a critical clinical symptom requiring thorough diagnostic evaluation, particularly due to its association with urothelial carcinoma. However, other differential diagnoses must also be considered, including renal carcinoma, infection, urolithiasis, and autoimmune disease.

We report the case of a 28-year-old male presenting with gross hematuria, flank pain, and dysuria. Imaging revealed bladder wall thickening and bilateral narrowing of the renal pelvis. Cystoscopy showed a large, polypoid lesion suggestive of urothelial carcinoma. Transurethral resection (TUR-B) was performed; however, histological analysis revealed granulation tissue with inflammatory infiltrates, multinucleated giant cells, and necrosis but no evidence of malignancy. Metagenomic sequencing identified *Peptoniphilus sp. SAHP1*, *Anaerococcus mediterraneensis*, and *Trichomonas vaginalis*, though their pathogenic role remained uncertain. Shortly after, the patient developed systemic inflammatory symptoms, including exanthema and gingivostomatitis. Biopsy of skin lesions showed leukocytoclastic vasculitis, and serologic testing yielded borderline myeloperoxidase-specific antineutrophil cytoplasmic antibody (MPO-ANCA) positivity. Under corticosteroid therapy, all symptoms, including the bladder lesion, regressed completely.

This case highlights a rare constellation of findings mimicking urothelial carcinoma, ultimately pointing to a probable autoimmune vasculitic process with possible secondary microbial colonization. It underscores the diagnostic challenges posed by atypical presentations and the need for integrative interpretation of clinical, histological, and molecular findings in complex cases.

## Introduction

Atraumatic gross hematuria should always prompt further diagnostic evaluation, as it represents a significant warning sign for various potentially serious conditions. The most critical differential diagnoses in this context include malignant tumors such as urothelial carcinoma and renal cell carcinoma [1-3]. The most common non-malignant differential diagnoses include ureterolithiasis [3,4] and hemorrhagic urinary tract infections [3,5]. IgA nephropathy [6] and lupus erythematosus may also cause macroscopic hematuria [3,7].

Vasculitis is a very rare underlying cause of hematuria. In most of these cases, antineutrophil cytoplasmic antibody (ANCA)-associated vasculitides involve the glomerular region of the kidneys and may result in glomerular macroscopic hematuria [8]. Involvement of the urinary bladder is rare and may occur either as part of a systemic vasculitic process or as an organ-limited manifestation. To date, very few case reports have described vasculitis affecting the urinary bladder [9,10]. To our knowledge, only two publications exist in which, during the evaluation of hematuria, a bladder tumor was observed macroscopically that could histologically be attributed to vasculitis [11,12].

There is currently no evidence supporting a causal relationship between acute urinary tract infections or nephrolithiasis and benign tumors.

## Case presentation

Initial presentation

We report a case of nonspecific, reactive granulomatous inflammatory tissue macroscopically mimicking urothelial carcinoma of the bladder but without any histological evidence of malignancy. To our knowledge, this is the first reported case of a benign tumorous lesion showing bacterial evidence despite clinical development suggesting an autoimmune origin and without specific histopathological confirmation.

A 28-year-old otherwise healthy male presented to the emergency department with flank pain, dysuria, and gross hematuria. He had no prior confirmed medical diagnoses, although he had experienced an episode of unexplained inflammatory syndrome affecting the shoulder and back 13 years earlier and had since undergone regular rheumatological checkups. There was no history of previous bladder procedures or trauma, and no family history of cancer.

As shown in Table 1, laboratory results showed mild signs of systemic inflammation, while urinalysis showed gross hematuria with other values not being analyzable. 

**Table 1:** Laboratory findings suggestive of a mild infection CRP: C-reactive protein; eGFR: estimated glomerular filtration rate

Test Name	Method	Result	Reference Range
CRP	Roche cobas PRO, immunoturbidimetric assay	44.3 mg/L	<5.0 mg/L
eGFR	CKD-EPI 2009 creatinine-based equation	119 mL/ min/ 1.73m2	>90 mL/ min/ 1.73m2
creatinine	Jaffe kinetic, rate-blanked	75 µmol/L	62-106 µmol/L
leukocytes	Automated impedeance/ flow cytometric method	6.11 Tsd/ µL	3.7 -11.2 Tsd/ µL
Spezific weight (Urine; Siemens-Stix Clinitek)	Siemens-Stix Clinitek	1.028 g/mL	1.005- 1.030 g/mL
Leukocytes (Urine)	Siemens-Stix Clinitek	limited by blood interference	negative
Nitrit (Urine)	Siemens-Stix Clinitek	limited by blood interference	negative
Intact and lysed erythrocytes (Urine)	Siemens-Stix Clinitek	limited by blood interference	<10 Ec/ µL

**Figure 1:**
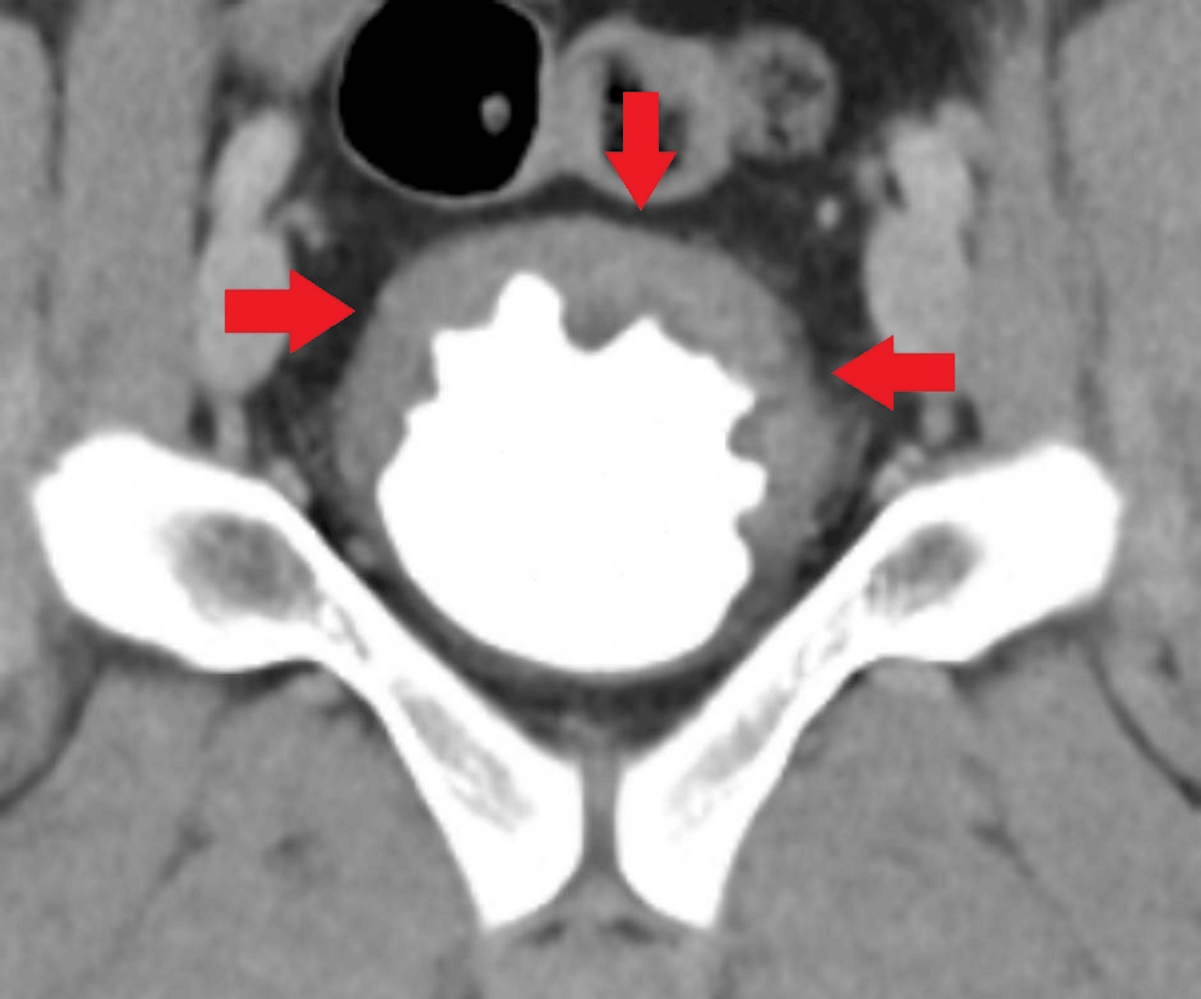
View of the partial bladder wall thickening in the coronal plane of a computed tomography scan, including the urographic delayed phase. Red arrows: affected area

As shown in Figure 1, a CT scan revealed thickening of the bladder wall in the region of the bladder base. There was no sign of ureterolithiasis, and the renal pelvis was narrow. As no definitive diagnosis could be established, the patient was discharged with symptomatic management and was referred to our outpatient clinic for further evaluation of the macroscopic hematuria. The urine culture showed no relevant bacterial growth. 

Diagnostic work-up

**Figure 2:**
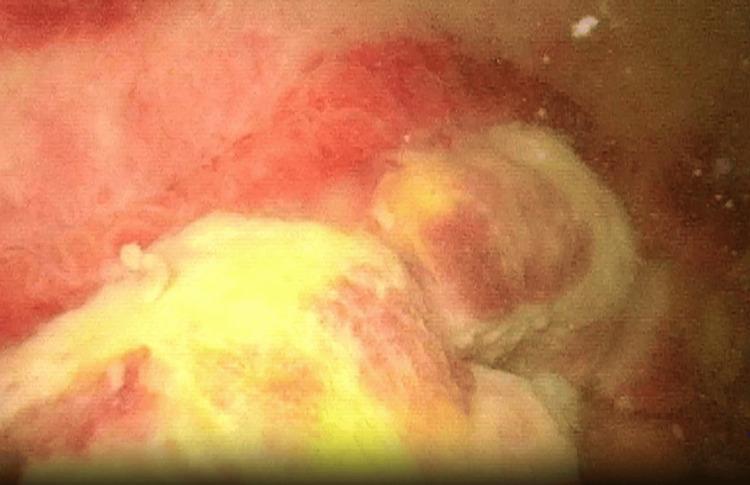
Cystoscopic view of part of the lesion

Six days after the initial presentation, the patient reported complete resolution of pain and dysuria. Intermittent macrohematuria persisted. Urethrocystoscopy revealed a bi-lobular configuration of the prostate. Intravesically, a widespread intravesical lesion involving the entire bladder base and extending bilaterally along the lateral walls was observed. The ureteral orifices remained unaffected. As shown in Figure 2, the lesion consisted of multiple nodular solid masses with papillary borders. Urinary cytology showed isolated atypical cells in the context of granulocytic inflammation.

Surgical findings

Based on the clinical presentation, particularly the cystoscopic appearance, there was a strong suspicion of urothelial carcinoma. Therefore, TUR-B was indicated. After identifying and marking the ureteral orifices, the lesion was resected. However, due to the extensive nature of the lesion, complete macroscopic resection could not be confidently achieved, corresponding to an oncological R2 resection. The underlying tissue appeared discolored, with a brownish-yellow hue. After two days of inpatient observation, the patient was discharged in good general condition.

**Figure 3:**
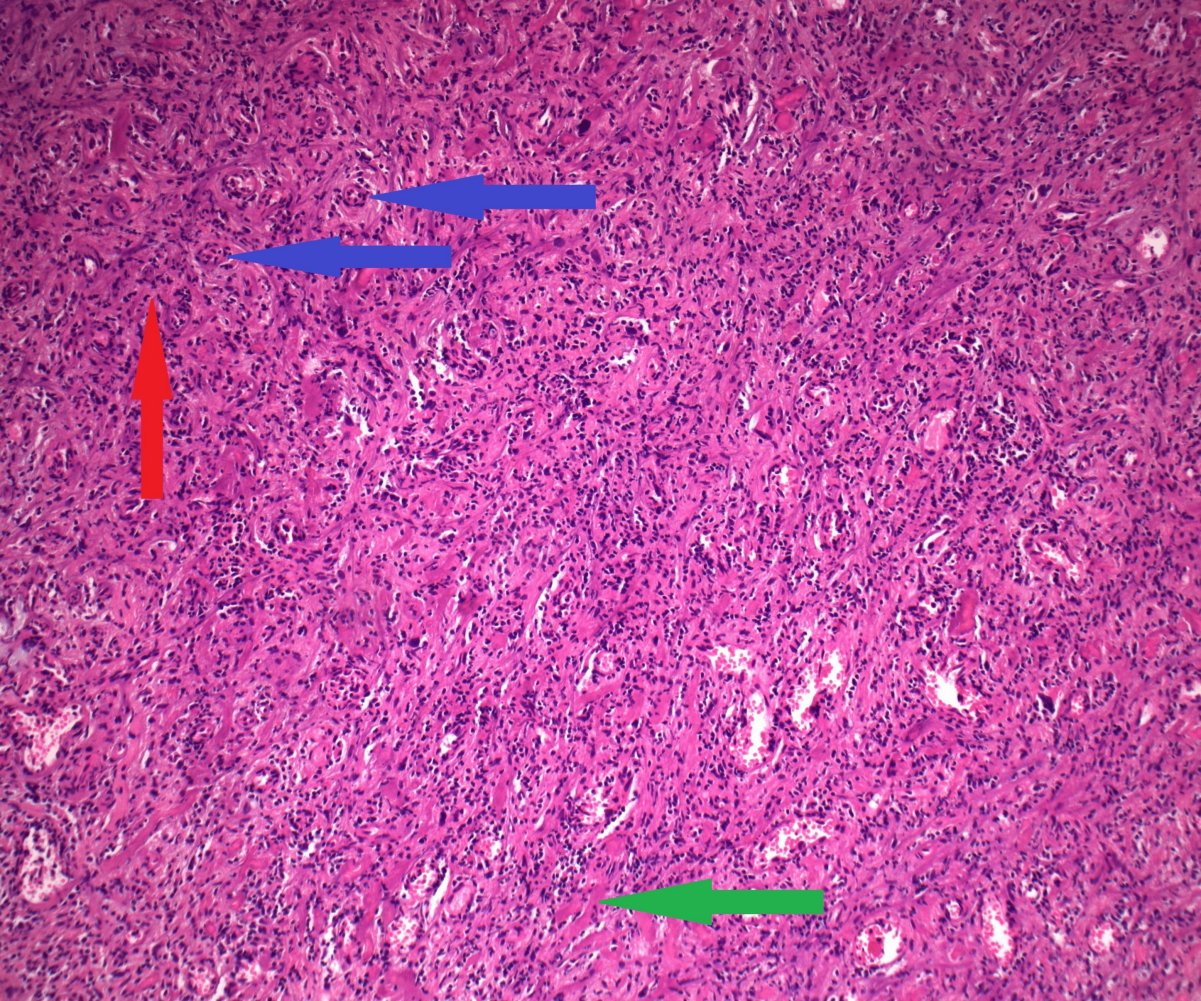
Histological section stained with hematoxylin and eosin (H&E) showing highly vascularized granulation tissue with numerous multinucleated cells at 100× magnification Red arrow: Area of granulation tissue; Blue arrows: Small vessels; Green arrow: Necrotic tissue

Histological examination of the sections of the bladder on HE stain revealed an ulcerated, highly vascularized, granulation tissue-like lesion of the bladder mucosa. It contained macrophage-lineage cells, some of which were multinucleated and showed mild reactive nuclear atypia, consistent with an inflammatory process. Interstitial and intravascular fibrinoid deposits contained structures consistent with bacterial colonies, which were detectable by Warthin-Starry silver staining. No organisms were demonstrated by Gram stain. Additionally, exposed necrotic tissue with Gram-positive bacterial colonization was noted. A representative section is shown in Figure 3. 

Metagenomic whole genome sequencing (mWGS) identified the organisms as *Peptoniphilus sp. SAHP1*, *Anaerococcus mediterraneensis*, and *Trichomonas vaginalis*.

Postoperative course

The patient developed novel systemic inflammatory manifestations, including a pustular and petechial exanthema localized to the gluteal, perianal, and proximal femoral regions, as well as a milder, generalized exanthema involving the trunk and facial cheeks. These dermatological findings were accompanied by marked fatigue and the onset of gingivostomatitis. All of these symptoms occurred de novo, with no prior history of similar presentations.

There was no evidence of an association between the bladder lesion and the newly developed symptoms. However, given a remote episode of unexplained inflammatory syndrome affecting the shoulder and back approximately 13 years prior, the patient’s treating rheumatologist was promptly consulted by the patient. In light of the new cutaneous findings, a skin biopsy was obtained from the gluteal region. Serologic testing for myeloperoxidase antibodies (MPO-ANCA) returned a borderline positive result, while other rheumatological antibodies were negative, as summarized in Table 2. 

**Table 2:** Laboratory findings from autoantibody testing c-/p ANCA: cytoplasmic/perinuclear anti-neutrophil cytoplasmic antibodies by immunofluorescence; PR3-ANCA: proteinase 3 antineutrophil cytoplasmic antibodies; MPO-ANCA: myeloperoxidase antineutrophil cytoplasmic antibodies

Test Name	Method	Result	Reference Range
c-/p ANCA	IIF (Titer)	>1:2560	<1:40
PR3-ANCA	ELISA	Neg.	< 3 U/mL
MPO-ANCA	ELISA	6 U/mL	< 5 U/mL

Histopathologic examination of the gluteal skin biopsy revealed features consistent with leukocytoclastic vasculitis. Owing to the nonspecific clinical presentation and the absence of any histological evidence, the bladder lesion and the cutaneous changes were still not considered to be related. Following the histological diagnosis and congruent laboratory findings, oral corticosteroid therapy was initiated, leading to a rapid initial improvement of most symptoms within a few days. After approximately three weeks of treatment, the cutaneous efflorescences still appeared to be mildly active, and subcutaneous methotrexate was introduced as an adjunct therapy. Approximately three months after the initiation of the therapy, only occasional mild skin rashes persisted. Six months after surgery and four months after the initiation of corticosteroid therapy, cystoscopy revealed an unremarkable bladder mucosa without evidence of a papillary tumor and with a resection site that was no longer clearly distinguishable. 

## Discussion

The literature search was conducted using PubMed and complemented by the artificial intelligence engine OpenEvidence. It revealed only two partially comparable case reports, all published more than a decade ago.

The first case, published in 2005, reports on a 59-year-old male polymorbid Australian smoker who presented with persistent irritative lower urinary tract symptoms accompanied by B-symptoms for months. Hematuria was not initially reported as a primary complaint but was later identified as microscopic hematuria during urinalysis. Computed tomography suggested a possible malignant process, and cystoscopy revealed abnormal mucosa but no presence of polypoid tumor growth. Histological examination confirmed the diagnosis of necrotizing vasculitis. A systemic vasculitis workup yielded negative results, and there was no mention of microbiological testing. The patient’s symptoms resolved within a few days following corticosteroid therapy [11].

The other case, reported in France, described a 31-year-old male and otherwise healthy smoker who, similar to our case, presented to the emergency department with acute pelvic pain accompanied by macroscopic hematuria. Clinical and laboratory findings initially pointed toward an infectious etiology, although both urine and blood cultures remained sterile. Imaging revealed focal bladder wall thickening and multiple pelvic thrombi. Cystoscopy identified a polypoid bladder lesion, which was completely resected. Histological analysis showed a non-granulomatous, small- and medium-vessel thrombotic vasculitis. The therapeutic approach included anticoagulation and antibiotic therapy, leading to complete resolution of symptoms. Notably, corticosteroids were not administered, and no bacterial pathogens were detected [12].

In comparison to the aforementioned cases, many parallels can be seen; for example, all the cases describe male patients. However, many key features distinguish our case: Most importantly, specific findings in the bladder biopsy were absent. After the development of systemic symptoms, including cutaneous presentation of new lesions, vasculitis was confirmed on another location of the body. Also, we were able to confirm the presence of bacteria, and the lesion resolved completely under immunosuppressive therapy.

Whilst histological evaluation revealed no evidence of malignancy, the presentation of the clinical course and constellation of findings invite further discussion and may open the door for new hypotheses regarding the underlying pathophysiology: Although the histomorphology and microbiological findings were compatible with an infectious etiology, the patient’s medical history and clinical course, most notably the complete resolution of the bladder lesion under immunosuppressive therapy, suggest an alternative underlying cause. 

Regarding a possible infectious etiology, we present the following thoughts: the presence of an ulcerated, highly vascularized, and granulation-like tissue formation typically occurs as part of tissue repair following mucosal injury or chronic irritation [13]. The presence of interstitial and intravascular fibrinoid deposits indicates localized vascular injury and fibrin leakage, changes commonly encountered in the context of acute-on-chronic inflammation and ulceration [14].

MWGS detected *Peptoniphilus sp. SAHP1*, *Anaerococcus mediterraneensis*, and *Trichomonas vaginalis* were found within the resected bladder lesion. While these findings initially raise suspicion of an infectious etiology, their clinical significance remains doubtful. This method is highly sensitive and prone to detecting background or contaminant microorganisms [15-18]. By choosing an access through the urethra, a natural entrance coated in mucosa, a similar type of bias seems even more plausible.

*Peptoniphilus* and *Anaerococcus* are anaerobic gram-positive cocci. Neither is addressed in the current EAU Guidelines on urogenital infections [5]. According to the literature, infection of necrotic tissue with *Peptoniphilus* in the context of diabetic foot syndrome may impede wound healing [19,20]. The organism has occasionally been isolated from the urine of asymptomatic individuals [21,22]. Nevertheless, its role in urinary tract infections, particularly in recurrent cases and particularly in immunocompromised patients or those with indwelling catheters, has not yet been clearly established. More likely, their detection reflects commensal colonization or contamination [23]. Trichomonas vaginalis is a flagellated protozoan and a known sexually transmitted pathogen. In women, primarily the vagina and urethra are affected by this pathogen [24]. In men, infection focuses on the urethra and prostate, with most infections remaining asymptomatic [25]. A bladder colonization with Trichomonas vaginalis has not been conclusively documented.

Conclusively, while microbial colonization may have occurred secondarily in necrotic tissue, an exclusively infectious etiology appears unlikely.

Second, we focus on a possible autoimmune and especially a vasculitic etiology: the patient developed systemic inflammatory symptoms postoperatively, including a pustular and petechial exanthema, gingivostomatitis, and profound fatigue. Histopathologic analysis of a skin biopsy revealed leukocytoclastic vasculitis, and serological testing showed borderline MPO-ANCA positivity. These findings are suggestive of an ANCA-associated small-vessel vasculitis presenting with cutaneous efflorescences and a systemic component. Although the absence of histological evidence for vasculitis in the bladder lesion represents a considerable limitation, the description of the specimen does not exclude the possibility of underlying vasculitis. In the context of vasculitis-related ulcerations, granulation tissue and necrotic areas are common findings even in the presence of active vasculitis [26]. 

Further immunosuppressive therapy led to complete regression of both systemic symptoms and the residual bladder lesion. The rapid and pronounced clinical improvement under immunosuppressive treatment strongly indicates that the lesion was immunologically driven rather than infectious in nature. This interpretation is strongly supported by the fact that, in infectious conditions, glucocorticoids and other immunosuppressive agents are generally contraindicated, as they are known to precipitate a rapid worsening of the disease [27].

Furthermore, the patient had a prior history of unexplained inflammatory joint pain during adolescence and was under rheumatological surveillance, suggesting a preexisting autoimmune diathesis.

Whether the bladder lesion represents a manifestation of the same underlying disease remains uncertain. Several explanations for the lack of histological evidence are conceivable: Possibly a sampling error occurred by missing out on critical tissue during R2 resection. Alternatively, a temporal dissociation seems possible, and histological signs have already resolved spontaneously by the time of resection [28]. Also, the bladder lesion may reflect a reactive process rather than a direct autoimmune target.

Finally, we present the possibility of a mixed etiology, taking all clinical, histological, and laboratory findings into consideration.

The temporal association between the onset of the bladder lesion, the systemic symptoms, and the histologically confirmed diagnosis in the cutaneous efflorescences provides a weak indication of a possible correlation among the various manifestations. This assumption is further supported by their concurrent resolution under therapy. As noted above, the histological findings are more suggestive of an infectious process. However, neither the patient's characteristics, the clinical presentation, nor the identified bacterial species are consistent with such an etiology. A vasculitic lesion may impair local immune defenses, potentially allowing atypical pathogens to colonize the affected area [26,29,30]. This interpretation is supported by the rapid resolution of bladder symptoms with symptomatic treatment, the presence of infection-like changes on histology, and the disappearance of all intravascular and cutaneous efflorescences under immunosuppressive therapy.

It remains unclear whether a primary asymptomatic colonization of the bladder triggered an exaggerated immune response or whether such colonization occurred secondarily in an opportunistic manner. Such interactions between infection and immune dysregulation are well described in other organ systems. For instance, microbial superantigens have been implicated in the pathogenesis of vasculitides [29,30]. Whether a similar mechanism contributed here remains speculative.

## Conclusions

This case contributes to the limited evidence on vasculitis of the urinary bladder by highlighting the importance of clinical findings even in complex diagnostics. This case illustrates the susceptibility of bladder vasculitic lesions to opportunistic microbial colonization and underscores the need for caution when interpreting positive mWGS findings, particularly in specimens obtained from potentially contaminated tissue and in the absence of corroborating culture data or clinical indicators of infection. The high analytical sensitivity of mWGS increases the likelihood of detecting microorganisms that may not be clinically relevant, which further emphasizes the importance of integrating molecular results with histopathologic, microbiologic, and clinical context.

Despite the limitation posed by the absence of histopathological confirmation, the clinical course, especially the favorable response to immunosuppressive therapy and the exclusion of alternative diagnoses such as infection, supports the presumptive diagnosis. Especially in young, otherwise healthy patients, after reasonably excluding malignancy, a therapeutic trial of corticosteroids may serve not only as a treatment modality but also as a diagnostic tool providing credible evidence in unclear inflammatory lesions. 
